# Wuqinxi Exercise Improves Hand Dexterity in Patients with Parkinson's Disease

**DOI:** 10.1155/2020/8352176

**Published:** 2020-10-29

**Authors:** Tian Wang, Guiping Xiao, Zhenlan Li, Kuncheng Jie, Mengyue Shen, Yan Jiang, Zhen Wang, Xiangrong Shi, Jie Zhuang

**Affiliations:** ^1^School of Kinesiology, Shanghai University of Sport, Shanghai 200438, China; ^2^College of Chinese Wushu, Shanghai University of Sport, Shanghai 200438, China; ^3^Institute for Healthy Aging, UNT Health Science Center, Fort Worth, Texas 76107, USA

## Abstract

**Objective:**

This study was designed to evaluate the effect of Wuqinxi after one session and 12-week intervention on hand dexterity in patients with Parkinson's disease (PD).

**Methods:**

Forty-six elderly participants with mild-to-moderate PD were randomly assigned to the groups trained with Wuqinxi (*n* = 23) or stretching (*n* = 23). All participants practiced 60 min session of either of these exercises, 2 sessions a week for 12 weeks in standing position. The score of Purdue Pegboard Test (PPT) and time for Soda Pop Test (SPT) were performed to assess hand dexterity and motor function along assessing the 39 items of Parkinson's Disease Questionnaire before and after 12-week interventions. In addition, the PPT scores were compared before vs. after one session of either of these two exercise modes.

**Results:**

Single session with either Wuqinxi or stretching exercise tended to improve PPT scores in PD patients. Furthermore, the improved SPT time was significant (*P* < 0.01) following 12-week training interventions with Wuqinxi (−1.32 ± 0.38 sec) or stretching (−0.89 ± 0.16 sec), which showed no group difference (*P*=0.734). However, only the participants in Wuqinxi group significantly improved the PPT scores of the dominant hand (+0.61 ± 1.34), both hand (+1.83 ± 3.13) and assemble (+2.04 ± 3.44) performance after 12-week training intervention. In parallel with improved hand dexterity and motor function, 12-week Wuqinxi training also significantly improved the patient's emotional wellbeing.

**Conclusion:**

The Wuqinxi intervention could be safely and effectively applied to improve hand dexterity following single-session exercise or 12-week training, which were accompanied by improved quality of life in patients with mild-to-moderate PD.

## 1. Introduction

Hand dexterity is as essential as walking ability or posture stabilization for maintaining independence and quality of daily living activities (DLA) [1,2]. Patients with Parkinson's disease (PD) often have impaired hand dexterity [3–5] even in the early stages of the disease. Manual tremor, rigidity, or bradykinesia impair not only normal DLA, such as dressing, feeding, grooming, typing, and/or writing, but also the manual function and performance for utilizing assistive devices, such as crutches and wheelchairs in PD patients [6]. Although dopaminergic treatment can improve some main symptoms of PD, such as tremor, rigidity, or bradykinesia, impaired manual dexterity may be less responsive to pharmacological intervention [7]. Physical therapy has been proved to be able to enhance PD patients' manual dexterity and hand function [8–10]. However, the question remains whether exercise training can be safely and effectively applied to improve hand dexterity in patients with PD.

Qigong exercise is a traditional Chinese physical activity that integrates body movements, breathing techniques, and meditation to manipulate the patient's energy (“qi”) to improve physical skill or achievement (“gong”), as well as mental and spiritual wellbeing [11]. Qigong is characterized by slow movement incorporated with moderated breathing, both of which help keep the mind in a state of calm relaxation [12]. It is believed that Tai Chi exercise [13] and Baduanjin Qigong [14] are safe and easy to practice by patients with mild-to-moderate PD. However, the impact of Wuqinxi exercise on hand dexterity of patients with PD has not been examined. Wuqinxi, also known as gymnastics of 5 animals (tiger, bear, deer, monkey, and bird), is a well-known traditional form of Qigong physical exercise [15, 16]. For a long time, Wuqinxi exercise has been established as a type of medical sport to provide physical wellbeing and to prevent and treat diseases in China. Documented reports indicate that Wuqinxi exercise has been applied to improve function of the lumbosacral multifidus [17], lumbar spine bone mineral density [18], immune function [15], and exercise capacity in patients with COPD [19] and to reduce the risk factors of cardiovascular disease and metabolic syndrome [20]. The focus of the present study was to test the hypothesis that Wuqinxi can be safely and effectively applied to improve hand dexterity in elderly adults with mild-to-moderate PD.

## 2. Materials and Methods

### 2.1. Study Participants

PD patients were recruited through Shanghai University of Sport or Xinhua Hospital, by referrals from neurologists and/or physical therapists, and distribution of the study information to local support groups for persons with PD. All participants provided an informed consent and signed the consent form which described the study procedure and was approved by the local ethical committee. The inclusion criteria for the study participants included a clinical diagnosis of PD, with a disease severity from mild to moderate level (rating from 1 to 3 out 5) according to the Hoehn and Yahr scale [21]; at least one limb with tremor, rigidity, bradykinesia, and/or postural instability based on the Unified Parkinson's Disease Rating Scale-Motor Examination (UPDRS-Part III) [22]; regularly taking prescribed medications for PD; ability to stand unaided and walk with or without an assistive device; willingness to be assigned to either of the two exercise groups; and an age of 55 to 80 years with medical clearance for participation in the study from his/her primary care physician. The exclusion criteria included currently involved in any behavioral or pharmacological intervention study or in instructor-led exercise training program, cognitive impairment based on medical history and/or clinical assessment, debilitating conditions or vision impairment that prevented from full participation in the study, and inability to attend to the exercise training program during the study period. Exercise training interventions started in September and completed in December 2018. A total of 58 patients gave an informed consent and passed physical examination. However, only 46 participants with mild-to-moderate PD enrolled were able to complete the study protocols. They were randomly assigned to the group trained with traditional Wuqinxi exercise (*N* = 23) or the group trained with the stretching exercise as control (*N* = 23) (see Table 1).

### 2.2. Exercise Interventions

#### 2.2.1. Wuqinxi Exercise

**Figure 1:**
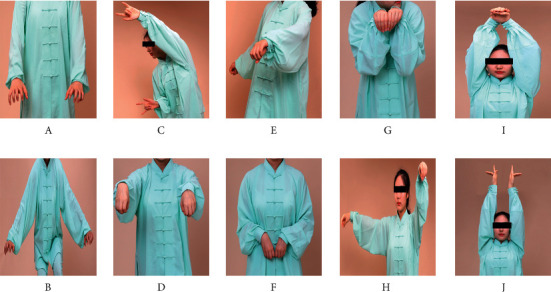
Illustrations of Wuqinxi exercise maneuvers. (a, b) Tiger exercise; (c, d) deer exercise; (e, f) bear exercise; (g, h) monkey exercise; (i, j) bird exercise.

Wuqinxi exercise (Figure 1) consisted of ten movements (two movements for each of the five animals): (1) tiger exercise (including raising the tiger's paws and tiger seizing the prey); (2) deer exercise (including colliding with the antlers and running like a deer); (3) bear exercise (including wobbling body like a bear and rubbing abdomen like a bear); (4) monkey exercise (including being on the alert like a monkey and plunking fruit like a monkey); and (5) bird exercise (including stretching arms upward like a bird and flying like a bird). These established traditional movements targeted to exercise muscles/joints including the facial muscles, eyes, mouth/teeth, hand/wrist, shoulder, cervical vertebra, and spine. Exercises of the fingers and toes are particularly emphasized for improving the blood circulation to the extremities but also for stimulating the acupuncture points that can be found there. Natural breathing was emphasized during the exercise.

#### 2.2.2. Stretching Exercise

Stretching exercise was intended to provide a variety of upper body and lower body movements with slow and gentle joint extension and flexion and trunk rotation. Natural breathing was also emphasized during the stretching exercise.

#### 2.2.3. Exercise Training

Both traditional Wuqinxi and stretching training programs were carried out 60 minutes per session, 2 sessions a week for 12 weeks. Both Wuqinxi and stretching exercises were performed in standing position. Background Health Qigong music was provided for all Wuqinxi and stretching training sessions. The participants' heart rates were monitored by Polar Team^2^ monitor (Polar Electro, Finland) during exercise training. During the initial 2 to 3 weeks of the training, each session mainly emphasized learning and practicing two or three maneuvers or movement forms through repetitions, along with practicing previously learned movements. All training sessions were led and supervised by a Qigong instructor during these initial weeks. All participants were able to learn and familiarize the complete set of the established Wuqinxi exercise or stretching movements within 3 weeks of the training program. The participants were requested to continue their prescribed medications and to maintain the daily routine, normal lifestyle, and diet during the entire period of the study.

### 2.3. Outcome Assessments

#### 2.3.1. Purdue Pegboard Test

The Purdue pegboard test (PPT) was used to test the timed speed and flexibility of hand movement, which has been commonly applied to evaluate hand dexterity in patients with PD [14, 23–25]. The test consisted of 4 subtests and the scores were determined by the number of pins placed in the pegboard within 30 seconds using the dominant hand (D), nondominant hand (ND), or both hands (B), and the number of pieces (containing pin, washer, and collar) assembled completely with two hands [25]. All tests were administered by a trained research assistant who followed the standard procedure and provided instructions according to the PPT manual. The test was administered individually for all participants. They all completed four PPT trials per test session. The PPT assessments were carried out before (i.e., baseline) and after 12-week training program (≥24 hours after the last exercise training session). In addition, all participants were tested before and after the first training session in the 4th week of the training program to assess the effect of one single-session exercise on the hand dexterity. They all were able smoothly to practice the complete set of the stretching exercise or the Wuqinxi exercise without reminder during the 4th week of the training.

#### 2.3.2. Soda Pop Test

The Soda Pop Test (SPT) was used to test the hand movements and hand-eye coordination [13]. The test performed on a cardboard platform (32″ in length and 5″ in wide) with six circles (3.25″ in diameter) drawn centered on the cardboard 1.5″ apart. Three full soda pop cans were used for the test and placed in every other circle starting from the side of the hand being tested. On the signal of “Go,” the stopwatch was started and the participant began to turn each can upside down into the adjacent empty circle within the drawn line, and then returned to the first can turned, replaced it in the original position, and continued with the other two cans. After two practice trials, the SPT process was repeated twice and the fastest time (in seconds) was documented as the test score. The dominant hand was used by all participants. The SPT measures were assessed and compared before (baseline) and after 12 weeks of Wuqinxi exercise or the stretching exercise training.

#### 2.3.3. Parkinson's Disease Questionnaire

All participants completed the 39 items of Parkinson's Disease Questionnaire (PDQ-39) [26, 27] before (baseline) and after 12-week exercise training. The PDG-39 set was categorized into 8 thematic domains: mobility (questions 1–10), activities of daily living (questions 11–16), emotional problems (questions 17–22), stigma (questions 23–26), social support (questions 27–29), cognition (questions 30–33), communication (questions 34–36), and bodily discomfort (questions 37–39). Every item of the PDQ-39 had a range from 0 to 4 up to a maximum of 156 points. The scores in each of these 8 domains and a total score of the 39 items were summarized and compared before and after 12 weeks of the exercise training interventions. A lower score indicated a better condition.

### 2.4. Statistics Analysis

Normal distribution of the data was examined using Shapiro–Wilk's test. Data before and after exercise interventions within the group were analyzed using paired *t*-test. Outcomes following exercise interventions between the two groups were analyzed by two-factor analysis of variance (ANOVA) to assess the significance of the group factor (i.e., the Wuqinxi exercise vs. the stretching groups) and the time factor (i.e., before vs. after exercise interventions). The change (Δ) in the measured variables, i.e., the outcome difference following the interventions between the two groups, was compared using *t*-test for two independent groups or Rank Sum test if the data failed to pass a normal distribution test. The statistical analysis was performed using SPSS 22.0 software. All data were reported as group mean ± standard deviation (SD) of the mean. *P* value < 0.05 was considered statistically significant.

## 3. Results

**Figure 2:**
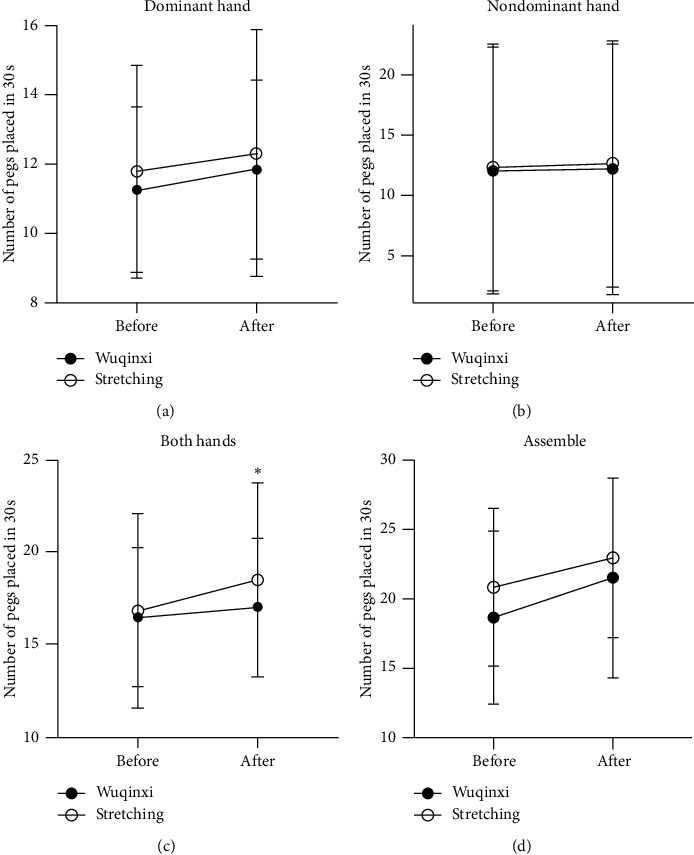
Placement of pegs after a single session of exercise. The number of pegs placed in 30 seconds after a single session of Wuqinxi (filled circles) and stretching (open circles). Asterisk denotes a significant difference between the groups. (a) Dominant hand. (b) Nondominant hand. (c) Both hands. (d) Assemble.

All patients safely tolerated the workload of practicing the traditional Wuqinxi or stretching exercise. There was not any unexpected or adverse event that occurred during the study.  Figure 2 shows the PPT performance following a single session of traditional Wuqinxi and stretching exercise. Both of these single-session interventions tended to improve the PPT scores significantly. There was no statistical significance for the group factor in the PPT score of the dominant hand (*P*=0.414), nondominant hand (*P*=0.061), or assemble (*P*=0.167), except the performance by both hands (*P*=0.001). The improved PPT score (Δ) from the two hand performance was greater or better (*P* < 0.001) following one session of stretching exercise (+1.65 ± 0.62) than Wuqinxi exercise (+0.52 ± 0.49).

**Figure 3:**
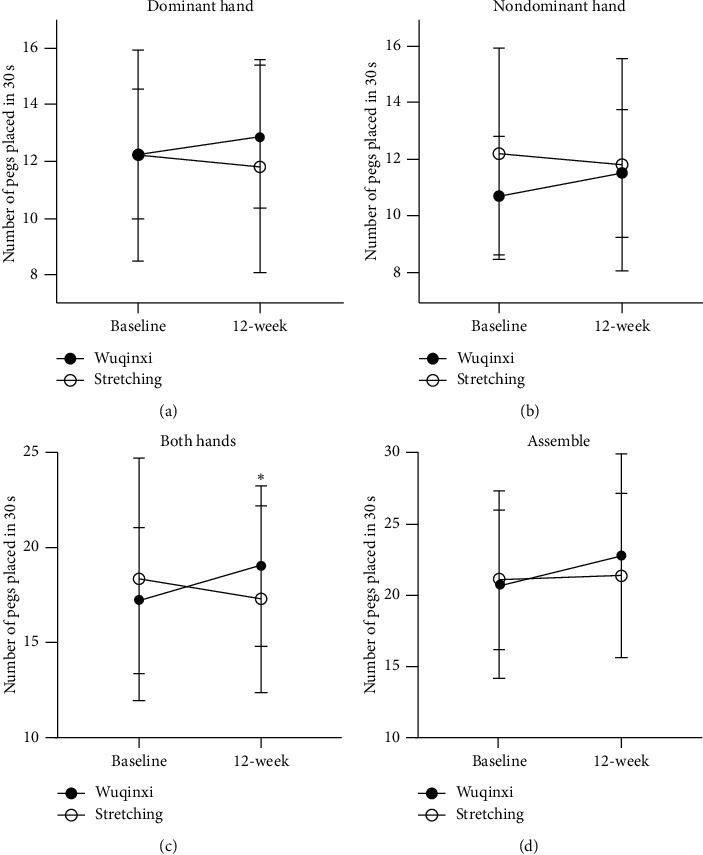
Placement of pegs after a 12-week training program. The number of pegs placed in 30 seconds in the Wuqinxi (filled circles) and stretching (open circles) groups after 12-week intervention. The PPT performance of the dominant hand (*P*=0.040), nondominant hand (*P*=0.056), both hands (*P*=0.010), and assemble (*P*=0.009) seem to be improved after 12-week training with Wuqinxi exercise. Asterisk denotes a significant difference between the groups. However, 12-week training with stretching exercise have no effect on hand dexterity tested in terms of the change in PPT performance. (a) Dominant hand. (b) Nondominant hand. (c) Both hands. (d) Assemble.

After 12-week training with Wuqinxi exercise, the PPT performance of the dominant hand (*P*=0.040), nondominant hand (*P*=0.056), both hands (*P*=0.010), and assemble (*P*=0.009) was improved (Figure 3). However, 12-week training with stretching exercise did not have the significant effect on hand dexterity tested in terms of the change in PPT performance. The improved scores from the dominant hand and both hand performances were significantly greater or better (*P*=0.003) after 12-week Wuqinxi training than stretching training (Figure 3).

**Figure 4:**
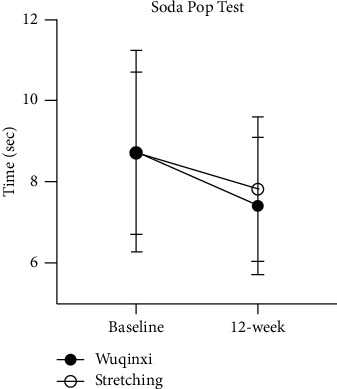
Soda Pop Test after a 12-week training program. The time spent on the Soda Pop Test is significantly improved in the Wuqinxi (filled circles) and stretching (open circles) groups after 12-week intervention. There is no difference between the groups.

The performance of the SPT was significantly (*P* < 0.01) improved following 12-week interventions with Wuqinxi exercise or stretching training (Figure 4). Nonetheless, the changes in the SPT scores were not significantly different between two interventions (*P*=0.657).

**Figure 5:**
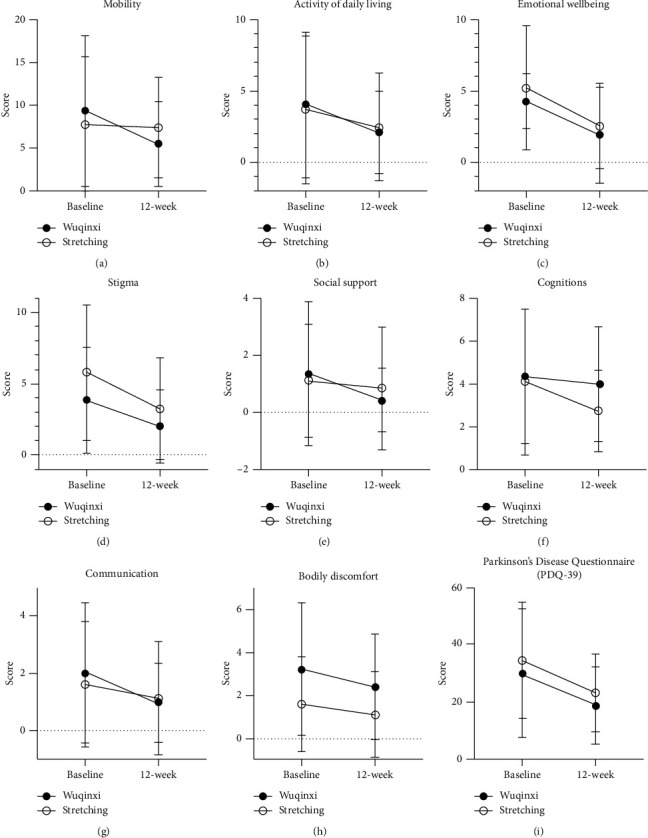
Scores of the 39 items of Parkinson's Disease Questionnaire before and after 12-week training. Scores of 8 dimensions and total points of the 39 items of Parkinson's Disease Questionnaire (PDQ-39) in the Wuqinxi (filled circles) and stretching (open circles) groups before vs. after 12-week exercise. Both interventions seem to improve the overall scores. The group difference is not significant. (a) Mobility. (b)Activity of daily living. (c) Emotional wellbeing. (d) Stigma. (e) Social support. (f) Cognitions. (g) Communication. (h) Bodily discomfort. (i) Parkinson's Disease Questionnaire (PDQ-39).

After 12-week training, both Wuqinxi (*P* < 0.01) and stretching exercise (*P* < 0.01) interventions significantly decreased (i.e., improved) the overall PDQ-39 scale scores according to ANOVA for the time factor (*P*=0.003). However, the improved scale scores were not different (*P*=0.92) between these two exercise interventions. Furthermore, both Wuqinxi exercise and stretching interventions similarly improved (*P* < 0.01) the scale scores in Domain II–Activities of daily living, Domain III–Emotional wellbeing, and Domain VII–Communication with no group difference in all these improved scores. The scale scores in Domain IV–Stigma and Domain VIII–Bodily discomfort showed the significance with the time factor following 12-week training intervention (Figure 5).

## 4. Discussion

The present study was the first to confirm that Wuqinxi exercises were safe to be applied on patients with PD and effective to help reduce the hand dyskinesia and non–gait freezing. The improved hand dexterity in terms of changed PPT scores was significant following one-session Wuqinxi or stretching exercise. After 12 weeks of exercise training, the improved hand function was more significant in the patients trained with traditional Wuqinxi than the participants in the stretching group. In alignment with the improved hand dexterity and movement which could potentially improve daily function and quality of life, the participants' emotional wellbeing and self-confidence seemed to be significantly improved following 12-week interventions with traditional Wuqinxi.

### 4.1. The Effect of Single-Session Exercise

Previously, Pelosin et al. [28] demonstrated that a single 45 min finger exercise could significantly improve finger motor performance. Mateos-Toset et al. [9] reported that hand dexterity and strength could be enhanced by one-session hand exercise in PD patients. In agreement with these previous studies, our data confirmed that following one 60 min single session of either traditional Wuqinxi or stretching exercise, hand dexterity could be significantly improved in the patients with mild-to-moderate PD (Figure 2). Both the dominant and nondominant hands had significantly improved PPT scores after single session of Wuqinxi exercise. Parkinson's disease is a progressive neurodegenerative disorder associated with the loss and/or dysfunction of dopaminergic neurons in the substantia nigra [29]. Thus, the acute effect of one-session exercise on the hand dexterity in PD patients was more likely a result of improved function of the skeletomuscular system, including muscles/joints of the fingers and palms/hands. Movements in Wuqinxi exercise target actions on the fingers, palm, and wrist, in addition to the movements of the facial muscles, eyes, teeth/mouth, fontanel, arm, shoulder, cervical vertebra, etc., according to 2003 Health Qigong Management Center of State General Administration of Sports in China. However, the acute effects of the 60 min single-session stretching exercise intervention also significantly improved performances by the nondominant hand and two hands in patients with mild-to-moderate PD. This seems to suggest that any finger and palm stretching can acutely and positively affect hand dexterity.

### 4.2. The Effect of 12-Week Training

Parkinson's disease is characterized by tremor, rigidity, bradykinesia, and/or postural instability, which impairs daily motor functioning and quality of life and, thus, affects both physical and mental status. Physical activity and/or exercise have been shown to retard the PD-related deterioration of motor functions and to prolong functional independence [30, 31]. The present study confirmed that a 12-week training program with Wuqinxi significantly improved hand dexterity, i.e., with reduced hand rigidity and bradykinesia in patients with mild-to-moderate PD, evidenced by improved PPT scores (see Figure 3) and reduced SPT time (Figure 4). However, 12-week training intervention with stretching exercise only significantly improved the SPT performance, not the PPT scores. It was not clear about this discrepancy or the difference in the PPT performance between 12-week training with Wuqinxi and stretching exercise or between a significant improved SPT performance and no change in PPT scores after 12-week training with the stretching exercise intervention. It might be due to an influence of seasonal circadian and/or ambient temperature which could negatively impact muscle metabolism [32] and physical functionality, especially in the elderly adults [33], since the baseline of the present study started in September and the 12-week training intervention ended in December 2018. It has been reported that 4 out of 5 animal imitation movements (except the tiger imitation) in Wuqinxi exercise, a well-known established traditional Qigong, could have a positive thermogenesis effect according to the increases in skin temperature [16]. However, exercise intervention induced thermogenesis in stretching exercise is unknown, which might be less significant than Wuqinxi exercise. Nonetheless, both 12-week training programs with Wuqinxi and stretching exercise seemed to be safe and to have beneficial influence in improving overall motor function (Figure 5) along with the improved hand dexterity evident by improved SPT scores.

### 4.3. Improvement of Emotional Wellbeing

It has been well recognized that patients with PD are often inflicted by depression or psychological commodities [34–36] because tremor, rigidity, bradykinesia, and/or postural instability impair daily activities as well as emotional state [37, 38]. Deficits in hand dexterity may limit or restrict the individual's capacity to complete basic life tasks, such as writing [39], personal hygiene [40], eating with chopsticks, and dressing. In alignment with the improvement of motor function and activities of daily living, our data demonstrated that both 12-week interventions with Wuqinxi and stretching exercise seemed to significantly improve emotional wellbeing and self-confidence in the elderly participants with mild-to-moderate PD, as evident by the improved scale scores in PDQ-39 Domain III–Emotional wellbeing and Domain VII–Communication (Figure 5). The present study was the first to demonstrate that improved hand dexterity and motor function was accompanied by significantly improved psychological or mental status in PD patients following Wuqinxi and stretching exercise interventions.

However, both interventions with Wuqinxi exercise or stretching exercise had no effect on PDQ-39 Domain VI–Cognitions (see Figure 5), which could be related to the fact that the participants in the present study had a normal cognitive function which provided no margin for further improvement.

### 4.4. Study Limitations and Perspectives

The main limitation of the present study was no sham-training or control group in the study. Future study should consider using crossover design, which allows to have a control group meanwhile all participants are able to partake the benefits of the exercise intervention. In addition, there was no follow-up assessment in the study to determine how long the intervention-induced benefits sustain after the termination of the training program. Assessing the effect of Wuqinxi on balance function should be the main focus of future study.

## 5. Conclusions

In conclusion, this study suggests that Wuqinxi exercise is safe, quick, and effective in improving hand dexterity in patients with mild-to-moderate PD. In association with improved physical motor function, the participants' emotional wellbeing and self-confidence seem to be enhanced following these exercise training interventions.

## Figures and Tables

**Table 1:** Characteristics of the study participants.

	Wuqinxi	Stretching
Number of participants	23 (10 women)	23 (13 women)
Age (yr)	67.0 ± 5.6	67.1 ± 6.1
Body mass index (kg/m2)	23.6 ± 2.6	23.2 ± 3.0
PD severity level		
1–1.5	9	9
2–2.5	12	12
3	2	2
Duration of disease (yr)	5.0 ± 3.1	5.3 ± 3.8

There is no statistical difference in any characteristic category between two groups.

## Data Availability

The data used to support the findings of this study are available from the corresponding author upon request.
